# 4-(2-Nitrobutyl)morpholin und 4,4′-(2-Ethyl-2-nitro-1,3-propandiyl)bismorpholin: MAK-Begründung, Nachtrag

**DOI:** 10.34865/mb222444kmxd10_2ad

**Published:** 2025-06-30

**Authors:** Andrea Hartwig, MAK Commission

**Affiliations:** 1 Institut für Angewandte Biowissenschaften. Abteilung Lebensmittelchemie und Toxikologie. Karlsruher Institut für Technologie (KIT) Adenauerring 20a, Geb. 50.41 76131 Karlsruhe Deutschland; 2 Ständige Senatskommission zur Prüfung gesundheitsschädlicher Arbeitsstoffe. Deutsche Forschungsgemeinschaft, Kennedyallee 40, 53175 Bonn, Deutschland. Weitere Informationen: Ständige Senatskommission zur Prüfung gesundheitsschädlicher Arbeitsstoffe | DFG

**Keywords:** 4-(2-Nitrobutyl)morpholin, 4,4′-(2-Ethyl-2-nitro-1,3-propandiyl)bismorpholin, Nase, Reizwirkung, Kanzerogenität, Formaldehydabspalter, Keimzellmutagenität, Sensibilisierung, 4-(2-Nitrobutyl)morpholine, 4,4′-(2-Ethyl-2-nitro-1,3-propanediyl)bismorpholine, nose, irritation, carcinogenicity, formaldehyde releaser, germ cell mutagenicity, sensitization

## Abstract

The German Senate Commission for the Investigation of Health Hazards of Chemical Compounds in the Work Area (MAK Commission) has re-evaluated 4-﻿(2-﻿ni﻿trobutyl)morpholine (70% w/w) and 4,4′-(2-ethyl-2-nitro-1,3-propanediyl)bismorpholine (20% w/w) [2224-44-4; 1854-23-5] (mixture) with regard to its carcinogenic﻿ity and germ cell mutagenicity classification, its ability to be absorbed through the skin, its sensitization potential and whether an occupational exposure limit value (maximum concentration at the workplace, MAK value) can be derived. Relevant studies were identified from a literature search. The mixture is a formaldehyde releaser and is expected to undergo rapid hydrolysis in aqueous solution. For this reason, the local irritation is attributed to the hydrolysis products formaldehyde, 1-nitropropane, and possibly 3-nitropentylmorpholine. Carcinogenicity, toxicity and genotoxicity of 4-(2-﻿nitrobutyl)morpholine and 4,4′-(2-ethyl-2-nitro-1,3-propanediyl)bismorpholine in the upper respiratory tract or nose, the likely target organs, have not been investigat﻿ed. The substance has clastogenic potential in vitro, presumably due to the release of formaldehyde. Formaldehyde was classified in Carcinogen Category 4 because it induces tumours in nasal tissues at concentrations that exceed their detoxification capacity. As a formaldehyde releas﻿er, 4-(2-nitrobutyl)morpholine and 4,4′-(2-ethyl-2-nitro-1,3-propanediyl)bismorpholine could be classified in Carcinogen Category 4. However, because it is not possible to derive a MAK value, the mixture has been assigned to Carcinogen Category 2 and given the footnote “Prerequisite for Category 4 in principle fulfilled, but insufficient data available for the establishment of a MAK or BAT value”. As there are no data for the systemic bioavailability and the formaldehyde that is released by hydrolysis in tissues, there is no experimental evidence that the formaldehyde reach﻿es the germ cells. Therefore, the mixture of 4-(2-nitrobutyl)morpholine and 4,4′-(2-eth﻿yl-2-nitro-1,3-propanediyl)bismorpholine has been classified in Category 3 B for germ cell mutagens. The mixture is skin sensitizing. Skin contact is not expected to contribute significantly to systemic toxicity.

**Table:** 

**MAK-Wert**	**–**
**Spitzenbegrenzung **	**–**
	
**Hautresorption**	**–**
**Sensibilisierende Wirkung (1993)**	**Sh**
**Krebserzeugende Wirkung (2024)**	**Kategorie 2^a)^**
**Fruchtschädigende Wirkung**	**–**
**Keimzellmutagene Wirkung (2024)**	**Kategorie 3 B**
	
**EKA**	**–**
	
Synonyma	**4-(2-Nitrobutyl)morpholin: ** *N*-(2-Nitrobutyl)morpholin **4,4′-(2-Ethyl-2-nitro-1,3-propandiyl)bismorpholin:** 4,4′-(2-Ethyl-2-nitrotrimethylen)dimorpholin 4,4′-(2-Ethyl-2-nitropropan-1,3-diyl)bismorpholin
Chemische Bezeichnung (IUPAC-Name)	4-(2-Nitrobutyl)morpholin 4-[2-(Morpholin-4-ylmethyl)-2-nitrobutyl]morpholin
CAS-Nr.	**4-(2-Nitrobutyl)morpholin:** 2224-44-4 **4,4′-(2-Ethyl-2-nitro-1,3-propandiyl)bismorpholin:** 1854-23-5
Formel	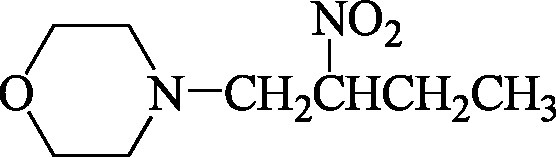 C_8_H_16_N_2_O_3_ 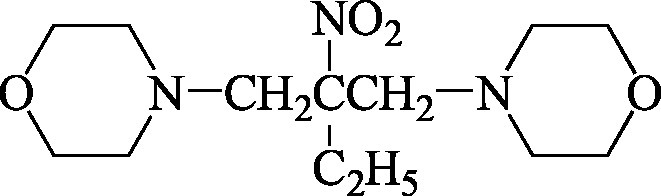 C_13_H_25_N_3_O_4_
Molmasse	**4-(2-Nitrobutyl)morpholin: **188,2 g/mol **4,4′-(2-Ethyl-2-nitro-1,3-propandiyl)bismorpholin:** 287,4 g/mol
Schmelzpunkt	ca. 10,5 °C (The Dow Chemical Company 2018)
Siedepunkt bei 1013 hPa	174,9 °C (The Dow Chemical Company 2018)
Dichte bei 25 °C	1,1 g/cm^3^ (The Dow Chemical Company 2018)
Dampfdruck bei 25 °C	0,0104 hPa (The Dow Chemical Company 2018)
log K_OW_	k. A.
Löslichkeit	11 g/l Wasser (Greim 1994)
pH-Wert	9,5**–**10 (The Dow Chemical Company 2018)
	
Hydrolysestabilität	bei pH-Werten < 6 Zerfall vorwiegend in Morpholin, Formaldehyd und 1-Nitropropan (Greim 1994), evtl. auch 3-Nitropentylmorpholin
Einsatzverbote	uneingeschränktes Verwendungsverbot seit 1993; siehe TRGS 611, GefStoffV, Verwendungsbeschränkungen für wassermischbare bzw. wassergemischte Kühlschmierstoffe, bei deren Einsatz N-Nitrosamine auftreten können (Hartwig und MAK Commission 2023 a) Das Gemisch ist im Rahmen von REACH lediglich vorregistriert (ECHA 2023).

^a)^ Voraussetzung für Kategorie 4 prinzipiell erfüllt, aber Daten für MAK- oder BAT-Wert-Ableitung nicht ausreichend

Hinweis: Nitrosaminbildner, Formaldehydabspalter, kann gleichzeitig als Dampf und Aerosol vorliegen

Es liegen eine Begründung (Greim 1994) und ein Nachtrag (Greim 2000 a) zur Spitzenbegrenzung vor. In diesem Nachtrag erfolgt eine Neubewertung der Daten zur Formaldehydabspaltung nach aktuellem Vorgehen der Kommission (DFG 2024). Unter diesem Aspekt werden in diesem Nachtrag nur die bewertungsrelevanten Endpunkte reevaluiert.

Die Begründung aus dem Jahr 1994 bezieht sich ausschließlich auf das Handelsprodukt Bioban^TM ^P-1487 bestehend aus 70 Gew.-% 4-(2-Nitrobutyl)morpholin, 20 Gew.-% 4,4′-(2-Ethyl-2-nitro-1,3-propandiyl)bismorpholin und 10 Gew.-﻿% inerten Bestandteilen. In wässrigen Lösungen von Bioban^TM ^P-1487 wurden N-Nitrosodiethanolamin und N-Nitrosomorpholin nachgewiesen, die als kanzerogen eingestuft sind (Greim 1994). Es liegen keine neuen Daten vor.

## Allgemeiner Wirkungscharakter

Das Gemisch aus 4-(2-Nitrobutyl)morpholin und 4,4′-(2-Ethyl-2-nitro-1,3-propandiyl)bismorpholin ist ein Formaldehydabspalter und Nitrosaminbildner. Der vermutlich kritische Effekt ist daher die kanzerogene Wirkung und lokale Reizwirkung von freigesetztem Formaldehyd am Atemtrakt. Das unverdünnte Gemisch wirkt an der Haut stark reizend, am Auge ätzend und es ist hautsensibilisierend.

Im Salmonella-Mutagenitätstest, im UDS-Test an primären Rattenhepatozyten und im Chromosomenaberrationstest an CHO-Zellen führt das Gemisch nicht zu genotoxischen Effekten. Ein TK^+/–^-Test in Mauslymphomzellen ist positiv. Ein In-﻿vivo/in-vitro-UDS-Test an Hepatozyten nach oraler Verabreichung des Gemisches an F344-Ratten ist ebenso negativ wie ein Maus-Mikronukleustest mit oraler Gabe.

Studien mit wiederholter inhalativer Gabe, Untersuchungen zur Reproduktionstoxizität und zur Kanzerogenität fehlen.

## Wirkungsmechanismus

Das Gemisch ist ein Formaldehydabspalter. Dies erklärt seine Reizwirkung und die genotoxische Wirkung im Maus-Lymphom-Test.

## Toxikokinetik und Metabolismus

4-(2-Nitrobutyl)morpholin ist hydrolytisch instabil mit einer Halbwertszeit von zehn Minuten, 4,4′-(2-Ethyl-2-nitro-1,3-propandiyl)bismorpholin hat Halbwertszeiten von 22 Stunden bei einem pH-Wert von 5, 45 Stunden bei pH 7 und 46 Stunden bei pH 9 (US EPA 2015). Es gibt keine experimentellen Untersuchungen zur dermalen Resorption von 4-﻿(2-﻿Ni﻿trobutyl)morpholin und 4,4′-(2-Ethyl-2-nitro-1,3-propandiyl)bismorpholin. Da für das Gemisch Angaben zu Molmasse und log K_OW_ fehlen, können Berechnungen mit Modellen nur anhand der Einzelsubstanzen erfolgen. Die mit Hilfe des EpiSuite-Programmes (US EPA 2022) berechneten log K_OW_-Werte betragen 0,18 für 4-(2-Nitrobutyl)morpholin und **–**0,54 für 4,4′-(2-Ethyl-2-nitro-1,3-propandiyl)bismorpholin.

In dem Gemisch sind 70 % 4-(2-Nitrobutyl)morpholin (M = 188,2 g/mol) und 20 % 4,4′-(2-Ethyl-2-nitro-1,3-propandiyl)bismorpholin (M = 287,4 g/mol) enthalten. Bei einer gesättigten wässrigen Lösung mit 11 g Gemisch/l Wasser entspricht dies ca. 7,7 g 4-(2-Nitrobutyl)morpholin und 2,2 g 4,4′-(2-Ethyl-2-nitro-1,3-propandiyl)bismorpholin (Greim 1994).

Eine gesättigte ca. 1%ige wässrige Lösung sollte aufgrund der hohen Verdünnung nicht mehr reizend wirken, da gemäß CLP-Verordnung (Europäisches Parlament und Europäischer Rat 2008) eine 5%ige Lösung eines Stoffs der Hautreizungskategorie 2 nicht als reizend einzustufen ist.

Modellrechnungen nach IH SkinPerm v2.04 (Tibaldi et al. 2014) und Fiserova-Bergerova et al. (1990) liefern für 4-﻿(2-﻿Ni﻿trobutyl)morpholin (7,7 g/l) unter Standardbedingungen (60 Minuten Expositionsdauer, 2000 cm^2^ exponierte Hautfläche) transdermale Fluxe von 1,6 bzw. 6,8 µg/cm^2^ und Stunde sowie Gesamtaufnahmen von 3,3 bzw. 13,6 mg. Die gleiche Berechnung ergibt für 4,4′-(2-Ethyl-2-nitro-1,3-propandiyl)bismorpholin (2,2 g/l) unter Standardbedingungen transdermale Fluxe von 0,07 bzw. 0,12 µg/cm^2^ und Stunde sowie Gesamtaufnahmen von 0,13 bzw. 0,24 mg. Nicht berücksichtigt wurde dabei eine zu vermutende rasche Hydrolyse des Gemisches im sauren Milieu der Hautoberfläche, die einer Resorption der unzersetzten Verbindungen entgegenwirkt.

## Tierexperimentelle Befunde und In-vitro-Untersuchungen

### Genotoxizität

#### In vitro

Im Salmonella-Mutagenitätstest, im UDS-Test an primären Rattenhepatozyten und in einem Chromosomenaberrati﻿onstest an CHO-Zellen führte das Gemisch bis zu zytotoxischen Konzentrationen weder mit noch ohne Zusatz metabolischer Aktivierung zu genotoxischen Effekten (Greim 1994).

In einem zweiten Mutagenitätstest mit dem Gemisch an den Salmonella-typhimurium-Stämmen TA98, TA100, TA1535 und TA1537 mit Konzentrationen von 0,007; 0,029; 0,12; 0,47; 1,88 und 7,5 mg/Platte mit und ohne Zusatz eines metabolischen Aktivierungssystems wurden bis zur höchsten Konzentration, die zytotoxisch wirkte, ebenfalls keine erhöhten Mutantenzahlen induziert (CalEPA 2010).

Ein TK^+/–^-Test in Mauslymphomzellen (L5178Y TK^+/–^) wurde mit Konzentrationen des Gemisches von bis zu maximal 4 µg/ml ohne und bis zu 9 µg/ml mit Zusatz eines metabolischen Aktivierungssystems durchgeführt. Höhere Konzentrationen waren zytotoxisch. Die Ergebnisse mit metabolischer Aktivierung zeigten einen konzentrationsabhängigen Anstieg der Mutationshäufigkeit (CalEPA 2010). Angaben, ob große oder kleine Kolonien gebildet wurden, fehlen.

#### In vivo

Ein In-vivo/In-vitro-UDS-Test mit dem Gemisch an Hepatozyten von je fünf bis sieben männlichen F344-Ratten, denen oral einmalig 0 (Maiskeimöl), 450 oder 900 mg/kg KG verabreicht wurden, verlief negativ. Die Positivkontrolle zeigte ein funktionierendes Testsystem an (CalEPA 2010).

Für einen Mikronukleustest mit dem Gemisch erhielten jeweils zehn (sechs in der Kontrolle) männliche CD-1-Mäuse pro Gruppe 0 (Maiskeimöl), 125, 250 oder 500 mg/kg KG an zwei aufeinanderfolgenden Tagen oral verabreicht. Die Untersuchung erfolgte 24 Stunden später. Für drei der Tiere wirkte die höchste Dosis letal, neun Tiere zeigten verminderte Aktivität, der prozentuale Anteil an PCE (polychromatische Erythrozyten) war vermindert. Es trat bei den exponierten Tieren im Vergleich zu den Kontrolltieren keine erhöhte Inzidenz an Mikronuklei auf. Die Positivkontrolle zeigte ein funktionierendes Testsystem an (CalEPA 2010). Im Hinblick auf die berichtete Instabilität der Testsubstanz im sauren pH-Bereich kann nicht ausgeschlossen werden, dass diese bereits im Magen zerfiel und Formaldehyd dort abreagierte und das Knochenmark nicht erreichte.

### Kanzerogenität

Hierzu liegen keine Daten vor.

## Bewertung 

Kritische Effekte sind die kanzerogene und lokal reizende Wirkung des Hydrolyseprodukts Formaldehyd sowie die mögliche Nitrosaminbildung mit ebenfalls kanzerogener Wirkung.

**Krebserzeugende Wirkung. **Es liegen keine Untersuchungen der krebserzeugenden Wirkung für das Gemisch aus 4-(2-Nitrobutyl)morpholin und 4,4′-(2-Ethyl-2-nitro-1,3-propandiyl)bismorpholin vor. In dem Gemisch ist eine Nitro﻿sa﻿minbildung möglich. Nitrosamine wirken kanzerogen (Henschler 1987).

Das Gemisch ist ein Formaldehydabspalter. Die lokale Kanzerogenität des Hydrolyseprodukts Formaldehyd ist ausführlich dokumentiert (Greim 2000 b; Hartwig 2010). Formaldehyd ruft Nasentumoren hervor, jedoch erst bei Konzentrationen, die die Entgiftungskapazitäten des Nasengewebes überschreiten. Daher ist Formaldehyd in Kanzerogenitäts-Kategorie 4 eingestuft.

Aufgrund der lokalen kanzerogenen Wirkung von Formaldehyd könnte in Analogie zu Formaldehyd eine Einstufung in Kanzerogenitäts-Kategorie 4 erfolgen. Da jedoch kein MAK-Wert für das Gemisch aus 4-(2-Nitrobutyl)morpholin und 4,4′-(2-Ethyl-2-nitro-1,3-propandiyl)bismorpholin abgeleitet werden kann, wird es der Kanzerogenitäts-Kategorie 2 zugeordnet und erhält die Fußnote „Voraussetzung für Kategorie 4 prinzipiell erfüllt, aber Daten für MAK- oder BAT-Wert-Ableitung nicht ausreichend“.

**MAK-Wert. **Es liegen keine Inhalationsstudien mit dem Gemisch am Menschen oder am Tier vor, aus denen ein MAK-Wert abgeleitet werden kann.

Das Gemisch hydrolysiert zu Formaldehyd, Morpholin und 1-Nitropropan (Greim 1994). Die schnelle Hydrolyse von 4,4′-(2-Ethyl-2-nitro-1,3-propandiyl)bismorpholin ist nachgewiesen, der zweite Bestandteil zerfällt langsamer (siehe „Toxikokinetik und Metabolismus“).

Wegen des Fehlens genauer Daten wird als Worst-Case-Abschätzung davon ausgegangen, dass das Gemisch beim Auftreffen im Atemtrakt durch Hydrolyse sofort den gesamten Formaldehyd freisetzt und dessen Abbau langsamer erfolgt als seine Bildung. Wenn die Entgiftungskapazitäten des Nasengewebes für Formaldehyd überschritten werden, ist mit einer kanzerogenen Wirkung durch Formaldehyd zu rechnen.

Morpholin wirkt ätzend an der Kaninchenhaut und am Auge und bewirkt in einer chronischen Inhalationsstudie an Ratten ab 50 ml/m^3^ Nekrosen in den Nasenturbinaten (MAK-Wert 10 ml/m^3^) (Greim 1996). Der Dampfdruck von Morpholin beträgt 9,8 hPa bei 20,3 °C (ECHA 2022). 1-Nitropropan wirkt am Auge beim Menschen und bei Kaninchen leicht reizend und in einer Studie nach OECD-Prüfrichtlinie 422 stehen nach inhalativer Exposition von Ratten Degenerationen und Entzündungen des olfaktorischen Epithels und des Plattenepithels in der Nase ab 48 ml/m^3^ im Vordergrund (MAK-Wert 2 ml/m^3^). Der Dampfdruck beträgt 13,3 hPa bei 25 °C (Hartwig und MAK Commission 2017). Formaldehyd hat einen MAK-Wert von 0,3 ml/m^3^ (0,37 mg/m^3^), der vor der lokalen kanzerogenen Wirkung schützt und aus der augenreizenden Wirkung abgeleitet ist (Greim 2000 b; Hartwig 2010) und sein Dampfdruck bei 25 °C beträgt 5185 hPa (OECD 2002).

Der Dampfdruck des Handelsproduktes Bioban^TM ^P-1487 (0,0104 hPa, The Dow Chemical Company 2018) zeigt, dass es als Dampf-Aerosol-Gemisch vorliegen kann (Abschnitt I, DFG 2024).

Für Aerosole von Formaldehydabspaltern ist durch die Impaktierung im Atemtrakt mit einer stärkeren Wirkung als die durch dampfförmigen Formaldehyd verursachte zu rechnen. Dies wurde mit einem anderen Formaldehydabspalter, zu dem eine Inhalationsstudie vorliegt, gezeigt (siehe Begründung *N*,*N′*,*N′′*-Tris(β-hydroxyethyl)hexahydro-1,3,5-triazin; Hartwig und MAK Commission 2023 b).

Für das Gemisch aus 4-(2-Nitrobutyl)morpholin und 4,4′-(2-Ethyl-2-nitro-1,3-propandiyl)bismorpholin kann somit kein MAK-Wert festgesetzt werden.

Eine Spitzenbegrenzung und die Zuordnung zu einer Schwangerschaftsgruppe entfallen.

Bei Anwendung in verdünnten wässrigen Lösungen sollte mit einer vollständigen Hydrolyse gerechnet und daher der MAK-Wert für Formaldehyd (Greim 2000 b; Hartwig 2010) eingehalten werden.

**Keimzellmutagene Wirkung. **Im Salmonella-Mutagenitätstest, im UDS-Test an primären Rattenhepatozyten und im Chromosomenaberrationstest an CHO-Zellen führte das Gemisch nicht zu genotoxischen Effekten (CalEPA 2010; Greim 1994). Ein TK^+/–^-Test in Mauslymphomzellen zeigte einen konzentrationsabhängigen Anstieg der Mutationshäufigeit. Angaben, ob große oder kleine Kolonien gebildet wurden, fehlen. Ein In-vivo/In-vitro-UDS-Test an Hepatozyten nach oraler Verabreichung des Gemisches an F344-Ratten verlief ebenso negativ wie ein Maus-Mikronukleustest mit oraler Gabe (CalEPA 2010). Untersuchungen an Keimzellen liegen nicht vor.

Auch Formaldehyd zeigt nur mit hohen intraperitonealen Dosierungen positive Effekte im Maus-Mikronukleustest, nicht aber nach oraler Gabe (Greim 2000 b), sodass trotz der negativen oralen In-vivo-Tests eine keimzellmutagene Wirkung des Gemisches aus 4-(2-Nitrobutyl)morpholin und 4,4′-(2-Ethyl-2-nitro-1,3-propandiyl)bismorpholin nach Inhalation nicht völlig ausgeschlossen werden kann.

Formaldehyd ist in Kategorie 5 für Keimzellmutagene eingestuft. Dies bedeutet, dass bei Einhaltung des MAK-Wertes von 0,3 ml/m^3^ nur ein sehr geringer Beitrag zum genetischen Risiko für den Menschen zu erwarten ist (Greim 2000 b).

Das Gemisch aus 4-(2-Nitrobutyl)morpholin und 4,4′-(2-Ethyl-2-nitro-1,3-propandiyl)bismorpholin könnte zwar in Analogie zu Formaldehyd in die Kategorie 5 für Keimzellmutagene eingestuft werden, jedoch kann für das Gemisch kein MAK-Wert festgesetzt werden.

Da Daten zur systemischen Bioverfügbarkeit von 4-(2-Nitrobutyl)morpholin und 4,4′-(2-Ethyl-2-nitro-1,3-propandiy﻿l)bismorpholin und dem durch Hydrolyse freigesetzten Formaldehyd fehlen, liegt kein experimenteller Beleg vor, dass der freigesetzte Formaldehyd in aktiver Form die Keimzellen erreicht. Daher wird das Gemisch aus 4-(2-Nitrobutyl)morpholin und 4,4′-(2-Ethyl-2-nitro-1,3-propandiyl)bismorpholin in Kategorie 3 B für Keimzellmutagene eingestuft.

**Hautresorption. **Es liegen keine experimentellen Untersuchungen zur dermalen Resorption des Gemisches von 4-﻿(2-﻿Ni﻿trobutyl)morpholin und 4,4′-(2-Ethyl-2-nitro-1,3-propandiyl)bismorpholin vor. Es gibt keine Daten mit wiederholter Gabe, aus denen sich ein systemischer NOAEL ableiten lässt.

Modellrechnungen liefern für eine gesättigte 1,1%ige wässrige Lösung des Gemisches Gesamtaufnahmen von maximal 13,6 mg für 4-(2-Nitrobutyl)morpholin und 0,24 mg 4,4′-(2-Ethyl-2-nitro-1,3-propandiyl)bismorpholin (siehe Abschnitt „Toxikokinetik und Metabolismus“) unter Standardbedingungen.

Aus 4-(2-Nitrobutyl)morpholin kann ein Molekül Formaldehyd freigesetzt werden, dies entspricht bezogen auf die Mas﻿se 16 % (30,03 g/mol Formaldehyd / 188,2 g/mol 4-(2-Nitrobutyl)morpholin). Aus 4,4′-(2-Ethyl-2-nitro-1,3-propandi﻿y﻿l)bismorpholin können zwei Moleküle Formaldehyd freigesetzt werden, dies entspricht 21 % (2 × 30,03 g/mol Formaldehyd / 287,4 g/mol 4,4′-(2-Ethyl-2-nitro-1,3-propandiyl)bismorpholin).

Durch die beiden im Gemisch enthaltenen Substanzen können über einen Zeitraum von einer Stunde maximal 2,226 mg Formaldehyd/2000 cm^2^ ((13,6 mg × 0,16) + (0,24 mg × 0,21)) aufgenommen und freigesetzt werden. Demnach wären das etwa 46 µg/1,25 Minuten (mittlere Halbwertszeit des Formaldehyds im Blut; Kaden et al. 2010). Die transdermale Aufnahme in den letzten sechs Halbwertszeit-Intervallen führt demnach zu einer im Blut zirkulierenden Formaldehydmen﻿ge von (46 + 23 + 11,5 + 6 + 3 + 1,5) μg = 91 μg. Der physiologische bedingte Formaldehydspiegel im Blut des Menschen beträgt etwa 2–3 mg/l (10–15 mg in 5 Liter Blut, frei und reversibel gebunden) (Heck et al. 1985).

Aus 4-(2-Nitrobutyl)morpholin kann ein Molekül 1-Nitropropan gebildet werden. Dies entspricht 47 % (89,09 g/mol Nitropropan / 188,2 g/mol 4-(2-Nitrobutyl)morpholin). Aus der berechneten Aufnahme von 13,6 mg 4-(2-Nitrobuty﻿l)morpholin aus einer 1,1%igen Lösung des Gemisches ergibt sich eine dermal resorbierte Menge von 6,4 mg 1-﻿Ni﻿tropropan.

Die systemische NOAEC für 1-Nitropropan nach 47-tägiger täglicher Inhalation beträgt bei Ratten 48 ml/m^3^ (177 mg/m^3^). Daraus ergibt sich bei Extrapolation auf eine chronische Exposition und Übertragung auf den Menschen (177 mg/m^3^ × 10 m^3^ × 7 Tage / 5 Tage / 4 (Zeitextrapolation) / 2 (Übertragung Tier-Mensch) / 2 (erhöhtes Atemvolumen am Arbeitsplatz)) eine systemisch tolerable Menge von 155 mg (Hartwig und MAK Commission 2017). Im Vergleich dazu ist die durch das Gemisch über die Haut aufgenommene Menge an Nitropropan zu vernachlässigen.

Der zusätzliche berechnete Eintrag durch transdermal aufgenommenen Formaldehyd und 1-Nitropropan ist unbedeutend und es erfolgt weiterhin keine Markierung mit „H“.

**Sensibilisierende Wirkung. **Es gibt anhand der aktuellen Datenlage keine Notwendigkeit für eine Änderung der Markierung. Die Markierung mit „Sh“ wird beibehalten, es erfolgt weiterhin keine Markierung mit „Sa“.
